# Dr. Charles Shupe

**Published:** 1915-10

**Authors:** 


**Dr. Charles Shupe,** Buffalo 1877, died at his home at Attercliffe Station, Ont., May 6, from heart disease.

